# Supplementary material for: Decrease in Bone Formation and Bone Resorption during Intravenous Methylprednisolone Pulse Therapy in Patients with Graves’ Orbitopathy
Source: J Clin Med. 2022 Aug 26;11(17):5005. doi: 10.3390/jcm11175005 (PMC9457466; doi:10.3390/jcm11175005)
Supplement: Supplementary file 1 [file jcm-11-05005-s001.zip › jcm-1864366-supplementary.pdf]

SUPPLEMENTARY MATERIAL

Table S1. Correlations between bone turnover markers and selected parameters at baseline.

Variable	P1NP	CTX
Age	r = 0.15, *p* value = 0.51	r = 0.07, *p* value = 0.75
BMI	r = 0.07, *p* value = 0.77	r = –0.19, *p* value = 0.39
TRAB	r = –0.10, *p* value = 0.64	r = –0.21, *p* value = 0.34
TSH	r = 0.06, *p* value = 0.77	r = 0.39, *p* value = 0.07
fT4	r = –0.49, *p* value = 0.02	r = –0.32, *p* value = 0.13
fT3	r = 0.36, *p* value = 0.09	r = 0.44, *p* value = 0.17
25(OH)D	r = 0.40, *p* value = 0.25	r = 0.12, *p* value = 0.61
Lumbar BMD	r = –0.33, *p* value = 0.13	r = –0.06, *p* value = 0.80
Femoral neck BMD	r = –0.09, *p* value = 0.69	r = 0.07, *p* value = 0.75
TBS	r = 0.06, *p* value = 0.78	r = 0.34, *p* value = 0.13

P1NP, amino-terminal propeptide of type I procollagen; CTX, C-terminal telopeptide of type I collagen; BMI, body mass index; TRAB, thyrotropin receptor antibodies; TSH, thyroid stimulating hormone; fT4, thyroxine; fT3, triiodothyronine; 25(OH)D, 25-hydroxyvitamin D; BMD, bone mineral density; TBS, trabecular bone score
